# cGMP signaling regulates context-dependent sensory valence in *C. elegans*

**DOI:** 10.64898/2026.07.28.741361

**Published:** 2026-08-01

**Authors:** Ricardo F. Frausto, Navonil Banerjee, Michelle L. Castelletto, Ava E. Bignell, Breanna Walsh, Elissa A. Hallem

## Abstract

An animal’s response to chemosensory cues depends on the animal’s prior experience, internal state, or life stage. However, the molecular mechanisms that regulate sensory valence ( *i.e.,* whether a chemosensory cue is attractive or repulsive) remain poorly understood. We investigated the mechanisms that specify sensory valence using the responses of the free-living nematode *Caenorhabditis elegans* to carbon dioxide (CO _2_ ). *C. elegans* exhibits highly flexible responses to CO _2_ : well-fed animals are repelled by CO _2_ , while both starved animals and well-fed animals raised under high CO _2_ conditions are attracted to CO _2_ . Here, we show that CO _2_ attraction in animals raised at high CO _2_ requires a cGMP signaling pathway that involves the cGMP-dependent protein kinase EGL-4. This pathway does not regulate CO _2_ response in starved animals, indicating that the role of EGL-4 in mediating CO _2_ attraction depends on satiety state. Cultivation under high CO _2_ conditions leads to increased cGMP levels in the CO _2_ -detecting BAG neurons, consistent with a specific requirement for EGL-4 in high-CO _2_ -cultivated animals. We also show that EGL-4 regulates CO _2_ valence by altering neuropeptide expression in BAG. Our results indicate that sensory valence is established in a context-dependent manner at the level of the primary sensory neuron.

## Full Text Availability

The license terms selected by the author(s) for this preprint version do not permit archiving in PMC. The full text is available from the preprint server.

